# Physical activity and the ‘pediatric inactivity triad’ in children living with chronic kidney disease: a narrative review

**DOI:** 10.1177/20406223221109971

**Published:** 2022-07-16

**Authors:** Thomas J. Wilkinson, Lauren L. O’Mahoney, Patrick Highton, Joao L. Viana, Heitor S. Ribeiro, Courtney J. Lightfoot, Ffion Curtis, Kamlesh Khunti

**Affiliations:** NIHR Applied Research Collaboration East Midlands (ARC-EM), Leicester Diabetes Centre, University of Leicester, Leicester LE45PW, UK; NIHR Applied Research Collaboration East Midlands (ARC-EM), Leicester Diabetes Centre, University of Leicester, Leicester, UK; NIHR Applied Research Collaboration East Midlands (ARC-EM), Leicester Diabetes Centre, University of Leicester, Leicester, UK; Research Center in Sports Sciences, Health Sciences and Human Development (CIDESD), University of Maia, Maia, Portugal; Research Center in Sports Sciences, Health Sciences and Human Development (CIDESD), University of Maia, Maia, Portugal; Faculty of Physical Education, University of Brasília, Brasília, Brazil; Leicester Kidney Lifestyle Team, Department of Health Sciences, University of Leicester, Leicester, UK; NIHR Applied Research Collaboration East Midlands (ARC-EM), Leicester Diabetes Centre, University of Leicester, Leicester, UK; NIHR Applied Research Collaboration East Midlands (ARC-EM), Leicester Diabetes Centre, University of Leicester, Leicester, UK

**Keywords:** dynapenia, kidney disease, paediatric, physical activity, physical illiteracy

## Abstract

The ‘paediatric inactivity triad’ (PIT) framework consists of three complex inter-related conditions that influence physical inactivity and related health risks. In those living with chronic kidney disease (CKD), a multi-factorial milieu of components likely confound the PIT elements, resulting in a cycle of decreased physical functioning and reduced physical activity. In this review, we explore and summarize previous research on each of the three principal PIT components (exercise deficit disorder, dynapenia, and physical illiteracy) in the pediatric CKD population. We found those living with CKD are significantly physically inactive compared to their peers. Physical inactivity occurs early in the disease process and progressively gets worse as disease burden increases. Although physical activity appears to increase post-transplantation, it remains lower compared to healthy controls. There is limited evidence on interventions to increase physical activity behaviour in this population, and those that have attempted have had negligible effects. Studies reported profound reductions in muscle strength, physical performance, and cardiorespiratory fitness. A small number of exercise-based interventions have shown favourable improvements in physical function and cardiorespiratory fitness, although small sample sizes and methodological issues preclude the generalization of findings. Physical activity must be adapted and individualized to the needs and goals of the children, particularly those with acute and chronic medical needs as is the case in CKD, and further work is needed to define optimal interventions across the life course in this population if we aim to prevent physical activity declining further.

## Introduction

The onset of many chronic noncommunicable diseases may lie in early childhood,^1^ a critical period of development during which personal lifestyle choices and behaviour patterns are established, including the choice to have a physically active lifestyle.^2^ Physical inactivity, sedentary behaviour, and low muscle strength and cardiorespiratory fitness are strong determinants for the future development of many chronic diseases, with resulting morbidity and mortality.^3^ Physical inactivity is estimated to cause between 6% and 10% of major chronic diseases, such as heart disease and diabetes, and is responsible for ~9% of premature mortality, or >5 million deaths per year.^4^ The economic burden of physical inactivity is vast and has been conservatively estimated to cost healthcare systems international $ (INT$) 53.8 billion worldwide. In addition, physical inactivity–related deaths contribute to $13.7 billion in productivity losses and is responsible for 13.4 million disability-adjusted life-years worldwide.^3^

In children and adolescents, physical activity has been shown to benefit physical, mental, and social health, as well as academic performance.^5,6^ Nevertheless, the prevalence of physical inactivity is increasing during adolescence: 81% of adolescents aged 11–17 years are insufficiently active globally^2,7–9^ with physical activity declining by ~5% each year across childhood.^10^ Worryingly still is that the long-term adverse effects on physical inactivity of COVID-19 and lockdown restrictions have yet to be realized.^11,12^ Physically inactive children tend to become physically inactive adolescents who replace activity with sedentary behaviour. Janssen *et al.*^13^ found sedentary time increased by ~25% from ages 7 to 15 years with the largest changes in sedentary time occurring between 9 to 12 years, the period which spans the transition to secondary (high) school. Unsurprisingly, inactive adolescents often go on to become inactive adults.^2^ Physical inactivity in children and adolescents is a complex multi-factorial phenomenon that is influenced by a plethora of contributing factors. Understanding the complex interactions that contribute to physical inactivity is important to addressing this problem. Originally proposed as a tripartite framework,^14^ the ‘paediatric inactivity triad’ (PIT) is a condition observed in physically inactive youth involving three distinct yet inter-related components that drive physical inactivity: (1) exercise deficit disorder, (2) pediatric dynapenia, and (3) physical illiteracy. Understanding each of these components in isolation is important but collectively they present a ‘triple jeopardy’. Further understanding of how these components are influenced by socioecological factors is important to affecting their change and promptly initiating adequate interventions.^15^

**Figure 1.**
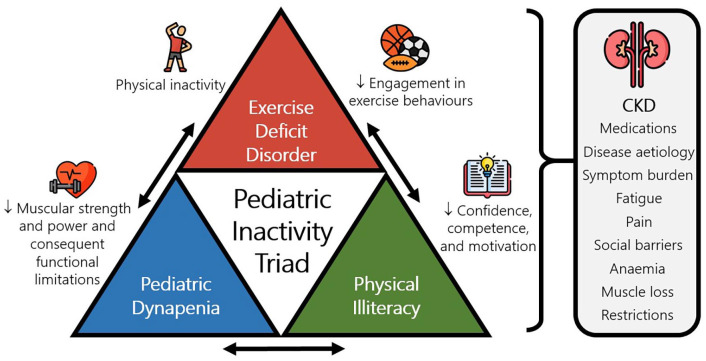
The ‘pediatric inactivity triad’ (PIT) framework consists of three inter-related conditions that influence physical inactivity and related health risks (exercise deficit disorder, dynapenia, and physical illiteracy). In chronic kidney disease (CKD), a multi-factorial milieu of components likely confounds the PIT elements that in turn cause a cycle of decreased physical functioning and reduced physical activity.

Chronic kidney disease (CKD) is a major health problem worldwide affecting around 9% of all adults.^16^ Data on the prevalence of CKD among children are scarce, although has an estimated prevalence of between 15 and 74.7 cases per million of the age-related population.^17^ Congenital anomalies of the kidney and urinary tract constitute the most common cause of paediatric CKD. However, although relatively uncommon in children, CKD can be a devastating illness with many long-term consequences and reduced life expectancy. Paediatric CKD, including disease etiology and cardiovascular complications, not only influence the child’s health, but also have long-term impact on the life of the adult that they will become.^18^ As with most long-term diseases, adults with CKD are woefully physically inactive^19^ and low levels of physical activity are associated with increased mortality and morbidity.^20,21^ Evidence for the beneficial role of physical activity in children and adolescents living with CKD is limited, and few studies have attempted to explore the complex interactions behind inactivity in this population. Given the adverse physiological, psychological, and social effects of CKD (Figure 1), it is likely that children and adolescents living with CKD have a greater risk of physical inactivity and may require specialist complex interventions to mitigate its effect. For example, children with end-stage kidney disease are often restricted from participation in exercise activities, especially those on haemodialysis (HD).^22^

In this review, we systematically explore and summarize previous research on each of the three principal components of the PIT in the paediatric CKD population, and suggest means to overcome the barriers associated with them. This review aims to expand our conceptual approach to physical inactivity in this population so to better prepare, identify, and treat paediatrics who are physically inactive.

### Systematic search methodology

To ensure we were reviewing the most appropriate literature, we systematically searched for literature pertaining to the PIT components in a paediatric CKD population. All etiology (e.g. chronic glomerulonephritis) and disease treatment modality (e.g. dialysis and transplantation) was included. We included all types of studies (e.g. RCTs, cross-sectional, cohort) if they contained relevant outcomes. We searched the following electronic databases from their date of establishment to December 2021: National Centre for Biotechnology Information (NCBI) PubMed and Clarivate Analytics Web of Science. Our search returned a total of 525 items (389 from NCBI PubMed and 136 from Web of Science). Fifty-eight duplicates were removed. This left 467 titles and abstracts to screen, and 401 were not suitable which left 66 full texts to be screened and extracted. Following full-text screening, a further 34 were removed. This left 32 articles that were included in this review (full search strategies and further detail can be found in Supplementary Material 1)

## Body

### Exercise deficit disorder

The first component of the PIT is ‘exercise deficit disorder’, defined as a condition characterized by levels of physical activity that are below the current recommendations.^7,15^ The term ‘exercise deficit disorder’ was originally chosen, above simply labelling someone as ‘inactive’, to emphasize the importance of this condition and to educate parents about the importance of daily physical activity.^14,15^

Research into physical activity in paediatric CKD populations is limited and most studies we identified reported on the physical activity and exercise behaviours of kidney transplant (KTx) recipients. Hamiwka *et al.*^23^ reported a group of 20 KTx recipients (mean age 14.3 years, 4.8 years post-KTx) were overall less physically active than a control group in total exercise minutes assessed using the Physical Activity Questionnaire for Children (PAQ-C) or Physical Activity Questionnaire for Adolescents (PAQ-A)^24^ (depending on age). Although the average step count per day was not significantly different from the control group, KTx patients were less physically active as measured by total self-reported exercise minutes [here, the number of exercise minutes that were not possible to measure with the pedometer (e.g. swimming, skiing, contact sports such as football) were self-recorded by participants]. Similarly, physical activity levels assessed *via* IPAQ were reportedly lower in 38 KTx patients (median age 13.6 years) compared to a non-CKD healthy group.^25^ A study from Italy^26^ showed 63% (10/16) of KTx recipients (mean age 16.0 years) had inadequate physical exercise levels, defined as <3 h/week. This was also significantly greater than a matched healthy control group. Levels of self-reported exercise behaviour were not associated with kidney function, although greater levels of physical activity were associated with greater cardiorespiratory fitness on a graded VO_2max_ test. In Wolf *et al.*,^27^ 101 KTx recipients were assessed >6 months post-transplant. Results showed physical activity levels (assessed by the PAQ-C or PAQ-A)^24^ were lower compared with controls. However, although 49% and 67% of respondents limited exercise before and after transplantation, respectively, 67% reported increased physical activity after transplantation. By parent report, healthcare provider advice included limiting certain activities (e.g. ball and contact sports and outdoor activities) (71%) due to concerns about kidney injury, but encouraging regular exercise (45%). These findings suggest patients and their parents perceive an emphasis on exercise limitations rather than the benefits of regular exercise and that perhaps interventions that encourage daily physical activity may be beneficial. Painter *et al.*^28^ found that in a sample of 25 paediatric KTx recipients (aged 15.7 years) and 15 dialysis (both HD and PD) patients (aged 16.4 years), *all participants* were physically inactive [assessed *via* a previous day physical activity recall (PDPAR)], with less than 10% of nonschool time being constituted of physical activity participation.

A study from the United States by Akber *et al.*^29^ in 44 patients across different CKD stages (stages 1–4, dialysis, KTx) found that physical activity levels, assessed by pedometer, *did not differ* among groups, although females were less active than males and physical activity was 44% lower among older children than younger participants. Lower levels of physical activity were associated with poorer physical performance and physical functioning, after adjustment for age, sex, and body mass index (BMI). In a follow-up study, participants in Akber *et al.*’s study^30^ underwent a 12-week pedometer-focused intervention to increase patients’ average daily step counts to the recommended targets for boys (15,000 steps/day) and girls (12,000 steps/day) over a 12-week study period. Overall, the intervention did not result in any meaningful changes in mean daily step count; however, closer examination of the different groups showed significant increases in daily step count for the KTx [100 steps/day (95% confidence interval [CI]: 14–208) and non-dialysis CKD (73 steps/day (95% CI: 115–262)] groups, while the dialysis group’s step count was decreased by 133 steps/day (95% CI: 325–58). Improvements in step count had favourable effects on exercise capacity and physical functioning. While this suggests a simple intervention may increase physical activity in some children and adolescents with CKD, it may not be suitable for those with more advanced disease staging and interventions may differ dependent on disease burden and staging.

In Johns *et al*.’s study,^31^ 10 adolescents (median age 18.0 years) underwent monthly group-based care sessions over 6 months. Sessions involved self-care activities (e.g. recording blood pressure) and educational discussion led by a nephrologist or dietician. While the primary clinical outcome was change in mean 24-hour ambulatory blood pressure, secondary outcomes included physical activity levels. Attendance was suboptimal, with only 40% of participants attending three or more sessions. However, the intervention did result in an increase in median steps per (waking) hour from 499 [interquartile range (IQR), 111–712] to 688 (IQR, 302–1107) at 6 months; however, this is still well below the paediatric hypertension guidelines recommendation of 12,000–15,000 steps per day.

El-Gamasy and Eldeeb^32^ used a validated Arabic-translated version of ‘The Child Behaviour Checklist’ to assess daily physical activities in 40 children aged 6–16 years undergoing HD. This study revealed that most children exhibited abnormal nutritional habits, disturbed sleep, decreased physical daily activities, impaired school achievement, changing emotions and behaviours, and depressed social relationships. For physical exercise, approximately 75% of the children engaged in no physical exercise with fear of injury and vascular access, school restrictions, tiring easily, and dyspnea given as the main barriers to engagement. Using data from the Chronic Kidney Disease in Children (CKiD) study in the United States, Clark *et al.*^33^ showed that in 224 aged 12–18 years with nondialysis CKD, only 13% of participants met recommendations for physical activity (defined as 30 minutes per day) compared to 25% of the reference group (taken from NHANES). No differences were seen between males and females. Furthermore, there was no difference between the number of days engaged in various time periods of self-reported physical activity (e.g. 20, 30, and 60 minutes), and after 1 year, the number of participants who met physical activity recommendations fell from 20 (14.7%) at baseline to 10 (7.5%). Fewer participants also reported playing on a sports team at follow-up.

Overall, several studies have explored levels of physical activity in paediatric CKD populations, although many of these are limited by small sample sizes, potential selection bias, and the reliance of self-reported physical activity behaviour. Some studies used pedometers to assess inactivity although there are limits to consider around the use of such devices given that (1) they are not validated in children with CKD and (2) they often rely on participants to self-report step counts without verification that pedometers are reset and worn at all times. In summary, the first component of the PIT is ‘exercise deficit disorder’ and, while limited by methodological considerations, consistent evidence suggests that paediatric CKD patients are severely more inactive than peers without CKD, and this inactivity is associated with poorer physical performance. No research has explored the longitudinal effect of physical activity behaviour in paediatric CKD patients on outcomes, such as disease progression and mental health, or in the case of KTx recipients, graft survival. More research is needed in those not requiring kidney replacement therapy, given the majority of evidence we found pertains to KTx patients, and only a handful of studies explored physical activity in dialysis patients. Furthermore, there have been limited efforts to implement interventions to increase physical activity behaviour, and those that have tried have had seemingly negligible effects.^30,31^ In this context, it is important to note that drivers of inactivity are likely different across disease sub-groups (e.g. in KTx recipients, barriers include fear of injury, while in those on dialysis, a lack of time may be a greater hindrance), and these should be accounted for in the promotion of physical activity behaviour.

### Paediatric dynapenia

The second PIT component, paediatric dynapenia, is defined as a condition characterized by low levels of muscular strength and power, and consequential functional limitations not caused by neurologic or muscular disease.^14,15^ This is an important constituent of physical activity behaviour as at a fundamental level, a sufficient amount of muscular strength and power is needed to move proficiently. Those with low levels of muscular strength are also more likely to remain inactive, experience functional limitations, and suffer activity-related injuries. Identifying at-risk children and adolescents may allow for better interventions designed to enhance muscular fitness.^14^

The most commonly used measure of strength employed in the kidney literature and clinical practice is that of handgrip strength (HGS). Advocated in nutritional guidelines as marker of malnutrition in adults,^34^ HGS is relatively simple to perform with several normative reference values available. Low HGS is a well-established predictor of mortality and risk of dialysis in adult CKD studies^35^ and recent efforts have attempted to standardize the protocol for HGS to ensure accurate and reliable data capture across research and clinical practice.^36^ Using the CKiD cohort, in 411 children with non-dialysis CKD, Hogan *et al.*^37^ found lower muscle strength (assessed by HGS) compared to healthy children, independent of age, sex, race, BMI, and other factors known for impacting muscle strength. In another study, 70% of children (51/73), who included a mixture of HD and non-dialysis CKD, showed HGS values below the 10th percentile for age and sex. No significant difference in HGS values were found between dialysis-dependent group and non-dialysis group, and HGS was found to be positively correlated to height, but not to lean tissue mass or serum albumin.^38^ In Tenbrock *et al.*,^39^ 30 children with a range of disease etiology had a significantly lower HGS than the normal control population, while Wolf *et al.*^27^ found the average grip strength *z* score of 101 KTx recipients was −1.1 and −0.7 in the right and left hand, respectively *versus* controls.

As well as muscular strength and power, give the inherent association with general physical functioning, we also looked at the wider physical dysfunction reported, including cardiorespiratory fitness and other physical-based performance measures. Cardiorespiratory fitness is defined as the ability of the circulatory and respiratory systems to supply oxygen during sustained physical activity and the ‘gold standard’ measure of cardiorespiratory fitness is attained through assessment of VO_2max_ (or VO_2peak_ as is often reported in functionally limited population groups). Cardiorespiratory fitness is an important aspect in addition to physical activity as it is considered to be an independent index predictive of mortality and is associated with a range of adverse health outcomes in many different chronic conditions, including adults with CKD.^20,40^ However, while the associations between cardiorespiratory fitness and outcomes in paediatric CKD populations in unknown, several studies have assessed cardiorespiratory fitness in this group.

In Sweden, Westphal Ladfors *et al.*^25^ found, compared to healthy controls, 38 KTx recipients (median age 13.6 years, 3.7 years post-KTx) had decreased exercise capacity measured by VO_2peak_ (34.5 *vs* 43.9 mL/kg/min, respectively). Lower exercise capacity was associated with higher fat mass index, lower physical activity, lower kidney function, and higher blood lipids. Interestingly, when participants were re-assessed annually over 3 years, *no change* in exercise capacity was observed. Lubrano *et al.*^26^ also found VO_2max/kg_ to be significantly higher in healthy controls with adequate physical activity levels than in the other groups (i.e. all KTx recipients and controls with inadequate levels of physical activity). Crucially, these data showed physically active transplanted children reached a fitness level comparable to sedentary healthy controls and better than that of sedentary transplanted children. In a study from Iran, Derakhshan *et al.*^41^ investigated exercise capacity in 44 KTx patients (mean age 16.0 years), comparing them to a healthy control group. The KTx patients achieved significantly lower maximal heart rate, maximal heart rate ratio, total energy expenditure, and VO_2max_ during the exercise than the control group. These paediatric KTx recipients also had severely impaired diastolic dysfunction.

In dialysis patients, Eijsermans *et al.*^42^ observed poorer fine motor skills (assessed *via* a Bruininks-Oseretsky test) and lower VO_2max_ values in 10 HD patients (aged 7–16 years) compared to an age-matched healthy reference group. Similarly, Schaar *et al.*^22^ reported that the cardiorespiratory fitness (VO_2peak_ assessed using cycle ergometer) of 14 HD patients (mean age 15.1 years) was significantly lower compared to a healthy age-matched population. This study also investigated changes pre- and post-dialysis with reductions in the time spent on the cycle ergospirometer, maximal workload, and VO_2peak_ seen post-dialysis. Pattaragarn *et al.*^43^ found that in 14 patients (both HD and PD), treadmill time, VO_2_, and VO_2AT_ were significantly lower in both groups of dialysis patients compared to a group of eight age-matched controls. While in a study of both paediatric KTx recipients (*n* = 25) and dialysis patients (*n* = 15), Painter *et al.*^28^ found there was no differences between groups for VO_2peak_ and quadriceps muscle strength measurements, although all values were below normative values. No improvement in any measures were observed from pre- to post-KTx in nine participants tested, except for a significant increase in percent fat, which may have negatively affected the change in muscle strength and VO_2peak_. Weaver *et al.*^44^ investigated VO_2max_ values in 46 children with a mixture of CKD stages (of which 22 were KTx recipients and 12 were undergoing HD). They found that VO_2max_ was similar between children with stage 2 CKD and controls, whereas lower VO_2max_ was observed among children with stage 3–4 CKD, those treated with HD, and KTx recipients. These data suggest that low VO_2max_ may already be present in children and adolescents with mild to moderate CKD and suggest that the cardiorespiratory system’s response to metabolic stress is attenuated early in the development of CKD. Like that seen elsewhere,^28^ no improvements were seen in the KTx recipients despite having good allograft function.

Graded laboratory-based cardiorespiratory fitness testing (e.g. assessment of VO_2peak_) is not always available, especially in pragmatic clinical or research settings. A common alternative is the 6-minute walk test (6MWT), which assesses the total distance walked in 6 minutes. While other field-based tests have been employed in older CKD populations (e.g. incremental shuttle walk test), the 6MWT was almost exclusively applied in the studies identified. Like assessment of VO_2peak_, all studies reported diminished 6MWT capacity. Alayli *et al.*^45^ studied the physical performance of 22 sedentary peritoneal dialysis (PD) patients (mean age 14.0 years, 33.9 months on dialysis), comparing them to 16 healthy children. The PD patients had a slower gait speed and poorer exercise capacity (assessed using the 6MWT) than the controls. They also found that quadriceps muscle strength was lower in PD patients. Although no difference in magnetic resonance imaging (MRI)-assessed muscle size was found, significant differences in T2 signal (marker of muscle quality) were observed. A study from the Netherlands by Takken *et al.*^46^ in 20 patients on dialysis (both HD and PD, mean age 14.1 years) found children with end-stage kidney disease had a reduced 6MWT performance (83% of predicted), irrespective of the reference values used. The strongest predictors of 6MWT performance were hematocrit and height, and 6MWT performance was not associated with VO_2peak_ (potentially raising some concern around the validity of both of these tests in this group), strength, or other anthropometric variables. No significant difference was reported in 6MWT performance between the patients on HD and those on PD. Also using the 6MWT, Watanabe *et al.*^47^ found that in 38 Brazilian patients (including PD, HD, and KTx patients, mean age 11.2 years), the median distance walked on the 6MWT was 538.5 (413–685) m, 84.1% of the reference value according to age, 90.6% according to age-corrected height, and 87.4% according to a predictive equation. Similarly, Akber *et al.*^29^ found the respective 6MWT distance in males and females was −2 and approximately −4 standard deviations below expected.

Given the, albeit small, data that cardiorespiratory fitness is diminished in children with CKD, it is clear interventions aiming to address this are important. While limited in quantity, several interventional trials have examined the effect of exercise on cardiorespiratory fitness outcomes. In a randomized control trial,^48^ 45 children/adolescent HD patients (mean age 14.5 years) underwent either thrice weekly intradialytic bicycle ergometer training or no training for 12-weeks. *No change* in dialysis adequacy or cardiorespiratory fitness (VO_2peak_) was found, although given the small sample size it is likely this study may be underpowered to detect such changes. Furthermore, the intensity of the intervention may have been too low to elicit physiological changes. Similar findings have been reported in adult HD patients following exercise.^49^ Participants in Van Bergen *et al.*^50^ underwent a 12-week, twice a week, graded community-based exercise programme supervised by local paediatric physiotherapists. The 50-minute sessions consisted of mixed aerobic and resistance training, but also added interval training in the last 4 weeks. In 20 children undergoing a either HD and PD, exercise capacity (assessed by VO_2peak_ and 6MWT) and muscle strength were improved following the exercise programme, but only in the five children who completed the training − 70% either did not start or did not complete the programme. Of these patients, seven did not complete or even start the exercise programme because of a combination of lack of time and motivational problems, and six were not able to continue because of medical problems.

Goldstein *et al.*^51^ and Goldstein and Montgomery^52^ found that in 10 children undergoing HD (median age 13.6 years), baseline exercise testing revealed a relatively *diminished* exercise capacity and functional status. Here, dynamometer-derived peak torque range, 6MWT distance, and upper extremity strength were all 50% lower than those reported for healthy controls. In the same study, a 3-month exercise programme (twice-weekly intradialytic exercise) *improved all tests of exercise capacity*, with 8/10 participants demonstrating improvements within two standard deviations of the mean values for healthy control populations. In Italy, a study of 10 HD patients (aged 15.3 years) found that a 3-month intradialytic exercise programme (30 minutes cycloergometer during the first half of the HD session performed two to three times a week) increased 6MWT performance (+4.9%), the number of repetitions during the sit-to-stand (STS)-60 second (+19%) and lower extremity strength (+29.3%).^53^ Previous study by this group^54^ had identified that 6MWT performance was associated with respiratory function (e.g. FVC, FEV1); however, no changes were seen in these parameters from the exercise programme. In Egypt, research from Abd-Elmonem *et al.*^55^ found that supervised progressive resistance exercise for 60 minutes, twice a week, for 6 months improved markers of quality of life and functional capacity in 32 children with CKD stage 3–4 (8–12 years old). Specifically, 6MWT performance increased by 98 m in the exercise group compared to the control group. Conversely, findings from a randomized control trial of 31 KTx recipients from Brazil (mean age 11.6 years), whom underwent home-based inspiratory muscle training for 6 weeks, showed improvements in predicted maximal inspiratory pressure, but *not in exercise capacity* assessed *via* the 6MWT.^56^

In summary, CKD (across all stages) is associated with impaired exercise capacity, physical performance, and muscle strength in children. Potential mediators of the impact of CKD on these outcomes are likely multi-factorial and include growth retardation, acidosis, poor nutritional status, and low physical activity.^37^ While some studies have shown improvements in physical function and cardiorespiratory fitness following successful exercise-based interventions, these studies are few and often of small sample size likely underpowered to detect any meaningful changes.

### Physical illiteracy

The third component of the PIT is physical illiteracy. This refers to the lack of confidence, competence, and motivation to engage in meaningful physical activities with interest and enthusiasm.^14,57^ Higher levels of physical literacy are recognized as foundational to regular participation in physical activity throughout the life course.^58^ Physical illiteracy encompasses psychomotor, cognitive, and affective domains of learning and as such interventions need to be reinforced with effective pedagogical, motivational, and social strategies so inactive youth can learn the value of physical activity.^57,59^ Research into physical illiteracy in paediatric CKD populations is severely lacking. Only a handful of studies have investigated components related to individuals to confidence, competence, and motivation to physical activity, and often these were assessed as part of a wider set of secondary outcomes. In particular, motivation towards physical activity was only described as barriers to engagement. Poor motivation in Goldstein and Montgomery^52^ meant that more than half of the children (on dialysis) initially enrolled did not complete the exercise intervention, with those receiving dialysis for <1 year more likely to complete the 3-month exercise programme. Similar findings were reported by Van Bergen *et al.*,^50^ where by 14/20 (70%) dialysis patients either did not start the programme or did not complete it due to lack of time or motivation, or medical problems. In this study, it was thought that patients would not engage with community-based programmes given leisure time is already significantly impacted by dialysis treatment.

Hamiwka *et al.*^23^ found that KTx recipients showed lower perceptions of sports competence compared to a control group. Although how this was explicitly assessed was unclear in their methodology, this is only inferred by the authors as KTx recipients reported participating in lower impact sports and activities. These findings also suggested this cohort of KTx recipients were not overly protected from physical play as they reported participation in many activities. However, it is possible that there are some activities that this patient group is more likely to choose than their healthy similar aged peers. Alternatively, these children might be offered or allowed by their parents and/or medical professionals to participate in activities with less physical contact. Clearly, the role of physical education teachers is important in engaging children with exercise and sporting activities. In 13 adolescents with CKD, Lee and Shin^60^ found that in the course of treatment, difficulties in physical activity, as well as the experience of a decrease in the desire for achievement, were found to limit the daily life of the participants. In this study, it was reported that notifying the physical education teacher about their condition was accurately conveyed and controlled without participating in excessive physical activity. In summary, given the dearth in evidence, a better understanding into how we can address and improve confidence, competence, and motivation to engage in physical activity is an important area of future research in CKD paediatric population.

### Summary of key findings

The PIT, a collection of three inter-related factors that drive physical inactivity in paediatric, embraces the complex etiology of physical inactivity. The inter-related components of the PIT, alone or in combination, pose significant health risks to children and adolescents, not just those with CKD. Our review shows that children and adolescents living with kidney disease are significantly physically inactive compared to their peers. Such inactivity appears to occur early in the disease process and progressively gets worse as disease burden and staging increases. In particular, children with end-stage kidney disease are often restricted from participation in exercise activities, and in some cases, less than 10% of non-school time involves some form of physical activity.^28^ Although physical activity appears to increase post-KTx (e.g. Wolf *et al.*^27^), it remains lower than healthy control cohorts. There have been limited studies on interventions to increase physical activity behaviour and those that have tried have had limited effects (e.g. Akber *et al.*^30^; interventions summarized in Table 1).

**Table 1.** Summary of interventions that aimed to increase physical activity or physical performance measures.

Study	Country	Population	Age (years)	Intervention	Key findings
Interventions aimed to increase physical activity					
Akber *et al.*^30^	USA	*N* = 44, CKD, dialysis, KTx	Mean: 15.1 ± 3.4	12-week pedometer-based intervention	KTx and CKD ↑ steps by 100 and 73 per day; dialysis ↓ 133 steps/day; ↑ in steps = ↑ physical performance (6MWT) and QoL (PedsQL)
Johns *et al.*^31^	USA	*N* = 10, CKD	Median: 18	Group-based care (monthly sessions over 6 months) inc. self-care activities and education	No change in self-reported satisfaction, QoL (PedsQL), sodium intake, up; small ↑ steps per waking hour (499–688)
Interventions aimed to increase physical performance or cardiorespiratory fitness					
Feldkötter *et al.*^48^	Germany	*N* = 54, HD	Mean: 14.5 ± 3.0	Thrice/weekly bicycle ergometer training or to no training during HD for 12-weeks	No change in dialysis adequacy (Kt/V), QoL (PedsQL), body composition, maximum power (Watts) or VO_2peak_
Van Bergen *et al.*^50^	The Netherlands	*N* = 20, HD, PD	Mean: 12.6 ± 3.3^a^	Twice/weekly community-based exercise programme for 12 weeks – both aerobic and resistance training	↑ VO_2peak_; ↑ maximum power (Watts); ↑ muscle strength; ↓ fatigue (CIS-20); no change in 6MWT or HRQoL (CHQ)
Goldstein and Montgomery^52^	USA	*N* = 10, HD	Median: 13.6	Twice/weekly intradialytic exercise programme (inc. upper/lower extremity biking, arm/leg weights) for 12 weeks	↑ lower extremity strength (different muscle groups); ↑ 6MWT; no change in HGS
Paglialonga *et al.*^53^	Italy	*N* = 10, HD	Median: 15.3	Twice or thrice/weekly intradialytic exercise programme for 12 weeks	↑ 6MWT; ↑chair stand test; ↑ lower extremity strength; no change in dietary, anthropometric, skinfold-thickness or cardiovascular indices
Abd-Elmonem *et al.*^55^	Egypt	*N* = 32, CKD	Mean:10.6 ± 1.3	Twice/weekly progressive resistance exercises for 24-weeks	↑6MWT; ↑ PedsQL
Carbonera *et al.*^56^	Brazil	*N* = 31, KTx	Mean: 11.6 ± 3.7^b^	Inspiratory muscle training	↑Aximal inspiratory pressure; no change in 6MWT

CKD, chronic kidney disease; KTx, kidney transplant recipient; HD, hemodialysis; PD, peritoneal dialysis; QoL, quality of life; PedsQL, paediatric quality of Life Inventory; CIS-20, checklist individual strength-20; CHQ, Child Health Questionnaire Parent Form 50; 6MWT, 6-minute walk test; HGS, Handgrip strength

a mean age for data for baseline and follow-up evaluation were available in five boys.

b Data from intervention group.

**Figure 2.**
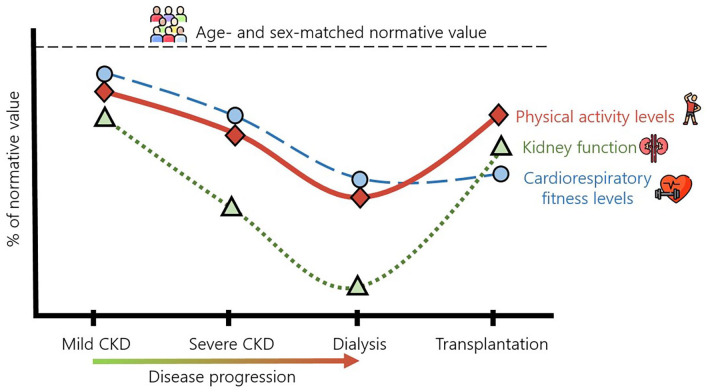
A complex change in physical activity and cardiorespiratory fitness occurs as disease etiology changes from stage to stage. Overall, declines in physical activity match negative changes in physical capacity as disease burden worsens. Transplantation seemingly has beneficial effects on activity levels but limited short-term changes on cardiorespiratory capabilities. CKD, chronic kidney disease.

The second PIT component refers to dynapenia, and studies in paediatric CKD populations have reported profound reductions in strength, physical function, and cardiorespiratory fitness (Figure 2). Like physical inactivity, physical attributes seemingly decline concurrently with disease progression. Strength values have been reported to be <10th percentile for age and sex for HD patients^37^ while exercise capacity is only ~80% of normative values.^46^ Worryingly, data suggests that low cardiorespiratory fitness is already present in children and adolescents with milder CKD staging,^44^ suggesting that the cardiorespiratory system’s response is reduced early in the development of CKD. Furthermore, limited improvements are seen in KTx recipients despite having good allograft function.^28^ Limited exercise-based interventions have shown favourable improvements in physical function and cardiorespiratory fitness (Table 1), but further research is needed to assess the effectiveness and implementation of different interventions in this population with adequate sample size and power.

### What next?

The low identified levels of physical function and fitness across all CKD stages may be the result of the disturbingly low levels of physical activity participation commonly observed. While the assessment of physical activity (e.g. often self-reported recall) limits definite interpretations of the findings, the consistently low levels should be of concern to the medical community caring for these patients. It is well known that the general population of children has decreased physical activity,^2,10^ so this problem is not necessarily unique to paediatric CKD patients. However, in children and adolescents with CKD, the increased cardiovascular risk from CKD, the reduced exercise capacity after HD initiation, and the excessive weight gain observed following transplantation must signal an effort on the part of the healthcare team to optimize physical functioning by providing education, counselling, and regular encouragement for regular physical activity participation. Healthcare professionals are ideally placed and suited to deliver influential messages promoting physical activity and behaviour change and every encounter represents an opportunity to ask about physical activity, provide advice, or signpost to appropriate pathways or opportunities.^2^

School settings also often provide an important structured means for children and adolescents to be active. It is well-established that children with CKD show impaired school achievement^32^ and that children living with CKD often feel they have disturbed school attendance, learning, social relation, and emotional well-being. In particular, dialysis treatment, which may prevent adequate schooling, may result in low academic achievement, poor learning, and fewer possibilities for entering into social relationships or establishing friendships in school, and this may explain a worse emotional state.^61^ Outside the CKD literature, interventions to increase children’s physical activity have had limited success and most physical activity programmes aimed at children and adolescents have been undertaken in a school setting,^62^ although there is limited evidence for this in CKD populations. Nonetheless, through appropriate input from educators, parents, and peers, children and adolescents with CKD should be encouraged to participate in regular physical activity at school.

### Designing programmes and interventions for children and adolescents living with CKD

Evidence suggests that physical inactivity, sedentary behavior, and poor cardiorespiratory and muscular fitness are strong risk factors for the development of chronic diseases. These result in significant morbidity and mortality risk and have great economic burden to the wider society. On the other end of the PIT continuum, is the paediatric activity triad (PAT). These components include adequate daily physical activity, physical literacy, and muscular fitness (i.e. strength, power, and endurance). Individuals may move from the PIT to the PAT, and within each triad move along the different arms of the spectrum at different rates and in different directions. Conversely, unlike PIT, the PAT confers significant benefits to the health and well-being of children. Regular physical activity, and higher levels of both cardiorespiratory and muscular fitness are associated with a reduction in development of many chronic diseases, and their concurrent morbidity and mortality.^3^ Alongside physical health, improving and normalizing the levels physical activity in those with CKD may also benefit mental and social health, as well as academic performance.^5,6^ Although there are different strategies for targeting inactive paediatric, the ultimate goal for every child is to remain in the PAT while making gains in each arm of that spectrum.^15^

While evidence continues to grow among the adult CKD population into the role and efficacy of engaging in physical activity, these interventions and programmes do not necessarily transfer to children with the condition. In addition to differences in physical size and physiological responses to exercise, in general, children are active in different ways and for different reasons than older populations. In order to develop a child-appropriate training concept, we must consider the everyday movements of a child, for example, short, fast movements in an aerobic state, similar to an interval training.^48^ Activity is often based around fun, making friends, and creating new games and challenges. As such informed interventions and motivation-enhancing, child-friendly training programmes are needed to facilitate participation in activities. This is where the art of designing and implementing youth exercise programmes comes into play. An understanding of paediatric exercise science needs to be balanced with an appreciation of teaching methods that motivate children to move and take responsibility for their own actions.^15^ Nonetheless, given that CKD is rare among children (between 15 and 74.7 cases per million of the age-related population)^17^ and may affect participation in physical activity, individualized interventions integrated with clinical care may be warranted.^30^ For example, considerations around disease management are important. Medications administered to KTx recipients, in particular immunosuppressive drugs, can limit exercise tolerance and physical activity.^40^ Sport participation may be a key element of physical activity in many children. However, sports are often severely impacted by living with CKD, specifically those on dialysis and KTx where particular activities may need to be reduced to protect fistulas or the transplanted organ.

### Limitations of this review

There are limitations to this review that should be considered when interpreting the findings. We used PIT as a novel basis to primarily conceptualize the inter-related association between physical activity and physical function. The three PIT components are largely observable characteristics resulting from more complex social, biological (e.g. maturity), and environmental determinants. In reality, multifaceted approaches are required to address these traits in this population. In the original PIT model, the three components are somewhat binary; for example, exercise deficit disorder is a ‘condition’ attained by not meeting physical activity guidance, where in reality physical activity encompasses a spectrum of behaviour including sedentary behaviour, sports, and light physical activity, which might be more appropriate targets in disease populations. Indeed in this review, we discuss more widely around the role of physical function, aside from just low muscle strength, and exercise, aside from just low physical activity. It may be that in CKD, a modified version of the PIT that encompasses more general physical activity and physical performance is useful. Although used intentionally for this purpose, the high medicalized terminology in the core constructs of the PIT may confuse or disconcert many people not familiar with these expressions. We included the entire range of ages across childhood so are unable to comment on the impact of maturation in this population.

## Conclusion

Physical inactivity and sedentary behaviour are strong risk factors for the development of chronic diseases with resulting morbidity and mortality, as well as economic burden to wider society from health and social care provision and reduced occupational productivity. Given that the majority of children and adolescents worldwide are not accumulating enough daily physical activity, innovative strategies are needed to activate inactive youth. In this review, we summarized the current literature against the PIT. This framework offers a collection of three inter-related factors that drive physical inactivity in youth and was designed to embrace the complex etiology of physical inactivity and offers a solution to better effect changes. We observed that paediatrics living across all stages of CKD face all components of the PIT and physical inactivity, physical dysfunction, and limited confidence and motivation are wide spread. It is apparent that resolute efforts from exercise and healthcare professionals, school administrators, public health officials, and others are needed, and these groups are in a unique position to prompt lifelong behaviour change. Physical activity and interventions must be adapted to needs and goals, particularly those with acute and chronic medical needs (e.g. CKD), and further work is needed to define optimal interventions across the life course in this population if we aim to prevent physical activity declining further.

## Supplemental Material

sj-docx-1-taj-10.1177_20406223221109971 – Supplemental material for Physical activity and the ‘pediatric inactivity triad’ in children living with chronic kidney disease: a narrative review Click here for additional data file. Supplemental material, sj-docx-1-taj-10.1177_20406223221109971 for Physical activity and the ‘pediatric inactivity triad’ in children living with chronic kidney disease: a narrative review by Thomas J. Wilkinson, Lauren L. O’Mahoney, Patrick Highton, Joao L. Viana, Heitor S. Ribeiro, Courtney J. Lightfoot, Ffion Curtis and Kamlesh Khunti in Therapeutic Advances in Chronic Disease

## Declarations

**Ethics approval and consent to participate:** Not applicable.

**Consent for publication:** Not applicable.

**Availability of data and materials:** Not applicable.
