# Supplementary material for: Physical activity and the ‘pediatric inactivity triad’ in children living with chronic kidney disease: a narrative review
Source: Ther Adv Chronic Dis. 2022 Jul 16;13:20406223221109971. doi: 10.1177/20406223221109971 (PMC9290151; doi:10.1177/20406223221109971)
Supplement: sj-docx-1-taj-10.1177_20406223221109971 – Supplemental material for Physical activity and the ‘pediatric inactivity triad’ in children living with chronic kidney disease: a narrative review [file sj-docx-1-taj-10.1177_20406223221109971.docx]

**Supplementary material**

**Supplementary material 1. Search methodology and results summary**

**Inclusion and exclusion**

We systematically searched for literature pertaining to the PIT components in a paediatric CKD population. Paediatric CKD defined as the presence of reduced renal function (presence of kidney damage, either structural or functional, *or* by a decline in glomerular filtration rate (GFR) below 60 mL/min/1.73m^2^ of body surface area for more than 3 months) in those *less than* 18 years of age. All aetiology (e.g., chronic glomerulonephritis) and disease treatment modality (e.g., dialysis, transplantation) was included. We included all types of studies (e.g., RCTs, cross-sectional, cohort) if they contained outcomes relevant to PIT. Interventional studies were included to explore possible means to improve PIT outcomes.

**Sources**

We searched the following electronic databases from their date of establishment to December 2021: National Centre for Biotechnology Information (NCBI) PubMed (which includes the Medical Literature Analysis and Retrieval System Online (MEDLINE)) and Clarivate Analytics Web of Science.

**Search terms**

The following key words formed the search strategies in each database: Physical activity; Exercise; Sport; Inactivity; Sedentary behaviour; Muscular strength; Dynapenia; Power; Physical function; Physical performance; Muscle function; Functional limitation; Disability; Confidence; Competence; Motivation; Knowledge.

The full search strategies for each database were as follows:

**Clarivate Analytics Web of Science (searched on 21/11/2021)**

((((((((((((((AK=(child*)) OR AK=(Adolescent*)) OR AB=(child*)) OR AB=(Adolescen*)) OR AB=(youth*)) OR AB=(Paediatric*)) OR AB=(Pediatric* )) OR AB=(child*))) OR AK=(child*)) OR TI=(youth*)) OR TI=(Paediatric*)) OR AK=(Paediatric*)) OR AK=(Pediatric*)) OR AB=(Pediatric*)

AND

((((((((((((((((((((AB=(exercise)) OR AK=(exercise))) OR AB=("Sedentary Behavior")) OR AB=(sport)) OR AB=("physical activity")) OR AK=("physical activity")) OR AK=("Sedentary lifestyle" )) OR AB=(MVPA)) OR AB=(Dynapenia)) OR AB=("Physical illiteracy" )) OR AB=("Pediatric inactivity triad ")) OR TI=("Pediatric inactivity triad" )) OR TI=("Exercise deficit disorder")) OR AB=("Exercise deficit disorder")) OR AB=(“Physical Functional Performance”)) OR TI=(“Physical Functional Performance”)) OR AB=("Muscle Strength" )) OR AB=("Cardiorespiratory Fitness" )) OR TI=("physical activity")) OR AB=("Sedentary lifestyle")

AND

(((((((((((((TI=("Kidney Disease*")) OR AB=("Kidney Disease*")) OR AK=("Kidney Disease*")) OR AK=(Dialysis )) OR AB=(Dialysis )) OR AB=(“Chronic Kidney Disease*”)) OR TI=(“Chronic Kidney Disease*”)) OR TI=(CKD)) OR AB=(CKD)) OR AB=(Hemodialysis )) OR AK=(Hemodialysis ))) OR TI=(CKD)) OR AK=(CKD)

**NCBI PubMed (searched on 15/11/2021)**

exp Child/

exp Adolescent/

Child*.tw.

Adolescen*.tw.

Infant.tw.

Youth.tw.

Paediatric*.tw.

Pediatric*.tw.

1 or 2 or 3 or 4 or 5 or 6 or 7 or 8

exp exercise/

exp Sedentary Behavior/

exp exercise therapy/

exp sport/

exercise training.tw.

physical activity.tw.

Sedentary Behavior.tw.

Sedentary lifestyle.tw.

exercise therapy.tw.

MVPA.tw.

sport.tw.

Dynapenia.tw.

Physical illiteracy.tw.

Pediatric inactivity triad.af.

Exercise deficit disorder.af.

exp Physical Functional Performance/

exp Muscle Strength/

exp Cardiorespiratory Fitness/

10 or 11 or 12 or 13 or 14 or 15 or 16 or 17 or 18 or 19 or 20 or 21 or 22 or 23 or 24 or 25 or 26 or 27

exp Kidney Diseases/

exp Renal Insufficiency/

exp Renal Dialysis/

Chronic Kidney Diseases.tw.

ckd.tw.

Renal Dialysis.tw.

kidney dialysis.tw.

Hemodialysis.tw.

29 or 30 or 31 or 32 or 33 or 34 or 35 or 36

9 and 28 and 37

**Screening**

References were exported to Endnote where titles and abstracts were screened independently by two reviewers. For those references that could not be excluded by title and abstracts full texts were obtained and screened by two reviewers.

**Results**

Our search returned a total of 525 items (389 from NCBI PubMed and 136 from Web of Science). Fifty-eight duplicates were removed. This left 467 titles and abstracts to screen, and 401 were not suitable which left 66 full texts to be screened and extracted. Following full text screening, a further 35 were removed. This left 31 articles that were included in this review. Out of the 31 papers included, four contained results pertaining to the exercise deficit disorder (EDD) component, two reported on EDD and pediatric dynapenia, 21 on pediatric dynapenia, one on pediatric dynapenia and physical illiteracy, and 3 on physical illiteracy. A PRISMA flow diagram is found below.


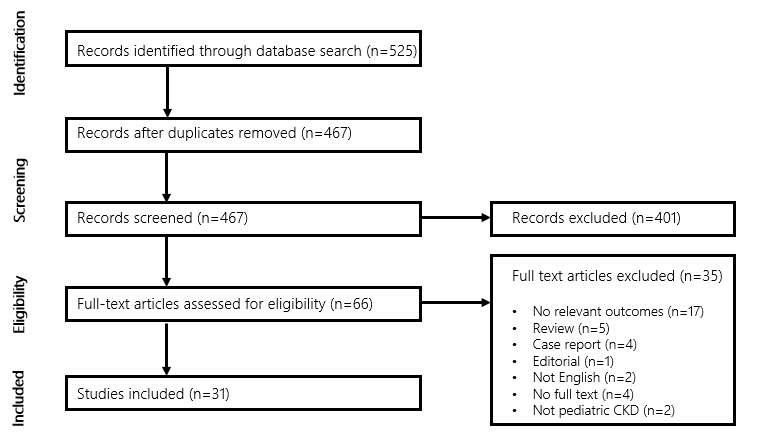


**Figure S1.** PRISMA flow diagram
